# Transcriptome-wide analysis links the short-term expression of the b isoforms of TIA proteins to protective proteostasis-mediated cell quiescence response

**DOI:** 10.1371/journal.pone.0208526

**Published:** 2018-12-11

**Authors:** Isabel Carrascoso, José Alcalde, Daniel Tabas-Madrid, Juan Carlos Oliveros, José M. Izquierdo

**Affiliations:** 1 Centro de Biología Molecular Severo Ochoa, Consejo Superior de Investigaciones Científicas, Universidad Autónoma de Madrid (CSIC/UAM), C/ Nicolás Cabrera, Madrid, Spain; 2 Centro Nacional de Biotecnología, Consejo Superior de Investigaciones Científicas, C/ Darwin, Madrid, Spain; University of Edinburgh, UNITED KINGDOM

## Abstract

Control of gene expression depends on genetics and environmental factors. The T-cell intracellular antigens T-cell intracellular antigen 1 (TIA1), TIA1-like/related protein (TIAL1/TIAR) and human antigen R (HuR/ELAVL1) are RNA-binding proteins that play crucial roles in regulating gene expression in both situations. This study used massive sequencing analysis to uncover molecular and functional mechanisms resulting from the short-time expression of the b isoforms of TIA1 and TIAR, and of HuR in HEK293 cells. Our gene profiling analysis identified several hundred differentially expressed genes (DEGs) and tens of alternative splicing events associated with TIA1b, TIARb and HuR overexpression. Gene ontology analysis revealed that the controlled expression of these proteins strongly influences the patterns of DEGs and RNA variants preferentially associated with development, reproduction, cell cycle, metabolism, autophagy and apoptosis. Mechanistically, TIA1b and TIARb isoforms display both common and differential effects on the regulation of gene expression, involving systematic perturbations of cell biosynthetic machineries (splicing and translation). The transcriptome outputs were validated using functional assays of the targeted cellular processes as well as expression analysis for selected genes. Collectively, our observations suggest that early TIA1b and TIARb expression operates to connect the regulatory crossroads to protective proteostasis responses associated with a survival quiescence phenotype.

## Introduction

T-cell intracellular antigen 1 (TIA1) and TIA1-like/related protein (TIAL1/TIAR) are RNA binding proteins (RBPs) with important roles in post-transcriptional gene regulation [1–3]. RBPs function both in the nucleus and the cytoplasm during every step of RNA metabolism to exert exquisite and specific control over gene expression [1–6]. Their regulatory roles are fulfilled at specific sites within the transcriptome through association with specific RNA sequence motifs (U-, UC- and AU-rich sequence stretches) [1–6]. In the nucleus, RBPs coordinate DNA-dependent transcription and processing of precursor RNAs (such as constitutive and alternative splicing) [4–6], whereas in the cytoplasm they guide RNA trafficking and stability as well as local mRNA translation [1–8].

Similarly, human antigen R (HuR/ELAVL1) is a ubiquitously expressed RBP with homology to the *Drosophila* ELAV (embryonic lethal abnormal vision) family, which modulates the nuclear and cytoplasmic fate of thousands of cellular RNAs [9]. Accordingly, HuR controls transcription, constitutive and alternative splicing, and also transports U- and AU-rich element-containing mRNAs from the nucleus to the cytoplasm [9–12]. Once in the cytoplasm, HuR directly regulates mRNA expression by either stabilizing mRNAs, influencing their translation, or interacting directly or indirectly with microRNAs and long non-coding RNAs [9–16].

All three RBPs play crucial roles in cell homeostasis by controlling the expression of critical genes involved in many biological programs including survival/death, proliferation/differentiation, inflammation, environmental stress and viral infections, among others, and are therefore important in human physiopathology [7, 8, 17–25]. They also have an essential function during embryogenesis as deficiency for TIA1, TIAR, or HuR (as well as ectopic over-expression of TIAR) in mice results in high rates of embryonic and postnatal lethality [26–29].

RNA sequencing (RNA-seq) is a powerful tool for the evaluation and quantification of transcriptomes and expression patterns in animals, tissues, cells or pathological conditions [30, 31]. Recent findings point to a link between TIA proteins and cell proliferation/differentiation [7], but the functions of TIA1 and TIAR in these processes are far from being understood. We therefore sought to characterize the gene expression patterns and biological processes important in defining cellular phenotypes during short-term overexpression of TIA1b, TIARb and HuR in HEK293 cells. Our findings provide new insights into the regulation of survival and quiescence networks mediated by TIA proteins.

## Materials and methods

### Cell culture

GFP-, GFP-TIA1b-, GFP-TIARb-, and GFP-HuR-expressing FT293 cell lines were generated using the Flp-In T-Rex System (Invitrogen) [17]. Immunocytochemistry was carried out as described [16, 17]. Murine embryonic fibroblasts (MEFs) knocked-out for TIA1 [26], TIAR [27], or EIF2AK2 [32] were maintained as described [33]. The lentiviral particles were generated from plasmids of second generation (pLKO series) that were transfected into HEK293T and the supernatants were collected. The shRNA against the human EIF2AK2 gene comes from SIGMA MISSION (# TRCN0000196400).

### RNA isolation

Total RNA was isolated with the RNeasy kit (Qiagen) and its quality was checked in an Agilent 2100 Bioanalyzer (RIN of 10) [17].

### Deep sequencing and genomic alignments

Three biological replicates for each sample type were deep-sequenced with Illumina technology (HiSeq Illumina platform, facilitated by the Centro Nacional de Análisis Genómicos [CNAG] from the Center for Genomic Regulation [CRG, Barcelona)]). FASTQ reads (strand-specific, pair-ends, 100 nucleotide lengths) were quality-checked with FASTQC (https://www.bioinformatics.babraham.ac.uk/projects/fastqc) and aligned against the human genome (GRCh38) with TopHat2 [34], using default options. Aligned reads, both left and right as well as pairs, were unique 90–92%, non-aligned 5.6–9.4% and discordant 0.38–0.52%. Overall, the results showed a good behavior of the reads during the alignment process and no adjustment or filter reading was performed. Sorting and indexing of alignment files (in BAM format) was carried out with SAMtools package [35]. The Integrative Genomic View (IGV) browser was used to visualize genomic alignments [36]. The data discussed in this publication have been deposited in NCBI's Gene Expression Omnibus (GEO) and are accessible through GEO Series accession number GSE113330.

### Differential expression of genes

Quantification of reads for each human gene (ENSEMBL annotation version GRCh38.82) was performed using the htseq-count function from HTSeq [37]. Data normalization and differential expression were calculated with the Bioconductor package DESeq2 [38] with the following options: cooksCutof = FALSE and independentFiltering = FALSE. P-values were adjusted using the FDR method [39]. Genes with logFC ≥ 1 or logFC ≤ -1, and FDR < 0.05 were selected as differentially expressed genes.

### Differential expression of exons

Exon-level (ENSEMBL annotation version GRCh38.82) reads were counted using dexseq_count.py script, included in DEXSeq [40] using settings for stranded-specific, pair-end reads. Data normalization and differential expression calculation of human exons were performed with DEXSeq with default options. Genes containing one or several exons with logFC > 1 or logFC < -1, *P* adjusted < 0.05 and exonBaseMean > 500 were selected as candidates for differential splicing analysis.

### Detecting alternative splicing events (ASE) with RNASeq-MATS

Multivariate analysis of transcript splicing (MATS) is a Bayesian statistical framework to detect differential alternative splicing events from RNAseq data. MATS can automatically detect and analyze alternative splicing events (ASE) corresponding to all major types of alternative splicing patterns. The differential ASE analysis was performed using RNASeq-MATS software [41–43]. Sequence data with multivariate analysis of transcript splicing (MATS) have been deposited in the European Nucleotide Archive (ENA) and are accessible through the ENA study accession number, PRJEB12377.

### Functional analysis of gene lists

GO database analysis was performed with the PANTHER Classification System (http://pantherdb.org). Statistical overrepresentation test was used to look for Biological Process GO term enrichment in significantly over- and under-expressed genes (*P* < 0.05) in FT293 cell lines. Venn diagrams were constructed using the Venny 2.1.0 tool (http://bioinfogp.cnb.csic.es/tools/venny).

### Cell proliferation, cell cycle and global translation analysis

Proliferation and cell cycle analysis of FT293 cell lines for 24–72 h after induction was performed as reported [17, 18]. For protein labeling, cells were incubated with methionine/cysteine-free DMEM supplemented with 3 μl of Easy Tag EXPRESS [^35^S] Protein Labeling mix (Perkin Elmer).

### Mitophagy, autophagy, and cell death

Mitophagy and autophagy were quantified using the fluorescence probes mKeima [44] and GFP-LC3-RFP [45, 46], respectively. Analysis of cell death was carried out as described [17, 18].

### qPCR analysis

Reverse transcription (RT) and real-time PCR was carried out at the Genomic PCR Core Facility at CBMSO. Analysis was performed on 2–3 independent samples in triplicate, including no-template and T-minus controls. Beta-actin (ACTB), glyceraldehyde phosphate dehydrogenase (GAPDH), and ribosomal RNA 18S were used as endogenous controls. Relative mRNA expression was calculated using the comparative cycle threshold method.

### Protein purification and western blotting

Cell lines were cultured and processed for protein isolation and western blotting as described [17, 18].

### Statistical analysis

The data were expressed as mean ± SEM. Student's t test was applied to determine statistical significance between 2 groups. *P* values < 0.05 were considered statistically significant.

## Results

**Fig 1:**
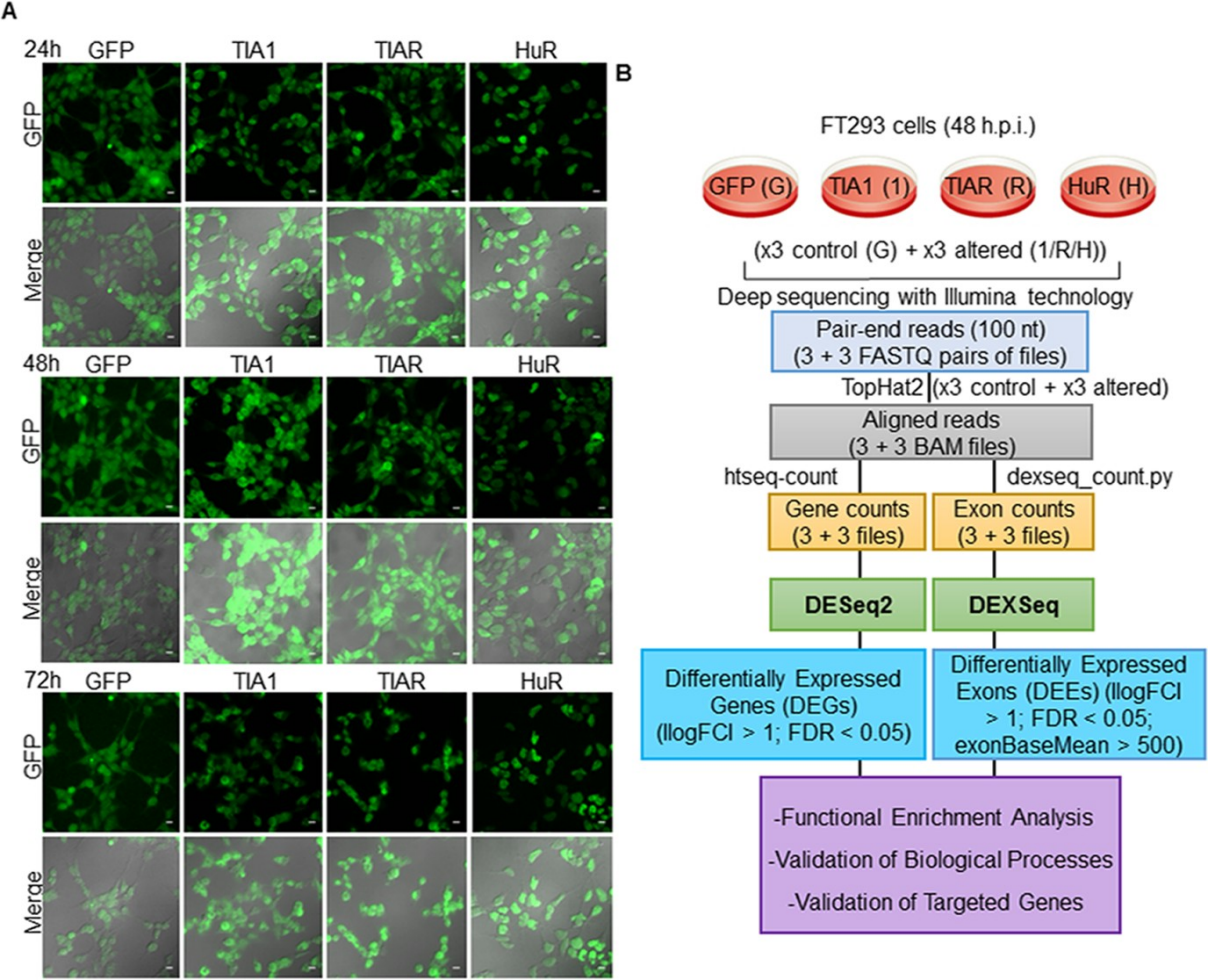
Characterization of the global transcriptomes of RBP-expressing FT293 cells by massive sequencing. (A) Expression patterns of GFP-tagged proteins. Fluorescence images together with corresponding phase contrast photographs (merge) are shown for 3 days. Scale bars represent 20 μm. (B) Working pipeline of the RNA-seq analysis. Workflow followed to analyze the datasets from massive-scale RNA sequencing. The legend identified as G, 1, R, and H indicates GFP, TIA1b, TIARb, and HuR samples, respectively.

We applied a massive sequencing approach based on RNA-seq to identify RNAs whose expression is modulated by TIA1b and TIARb isoforms and HuR. We first generated isogenic HEK293 cell lines (FT293) with inducible expression of GFP-tagged TIA1b, TIARb or HuR proteins, or a GFP-control, using the Flp-In T-Rex system [17], and examined their subcellular distribution at 24, 48 and 72 hours post-induction by fluorescence microscopy (Fig 1A). Results showed that the expression of the fusion proteins at 24–72 hours was consistent with the well-established subcellular patterns of nucleocytoplasmic and nuclear localization for endogenous TIA1 and TIAR, and HuR proteins, respectively; in agreement with our previous results [10, 17 (please, see details in S1 Fig), 18]. We therefore collected cells at 48 hours post-induction and processed them for RNA-seq analysis (Fig 1B and S1 Fig). A diagram of the sequencing workflow is shown in Fig 1B.

### Functional analysis of differentially expressed genes in TIA1b- and TIARb-expressing FT293 cells

**Fig 2:**
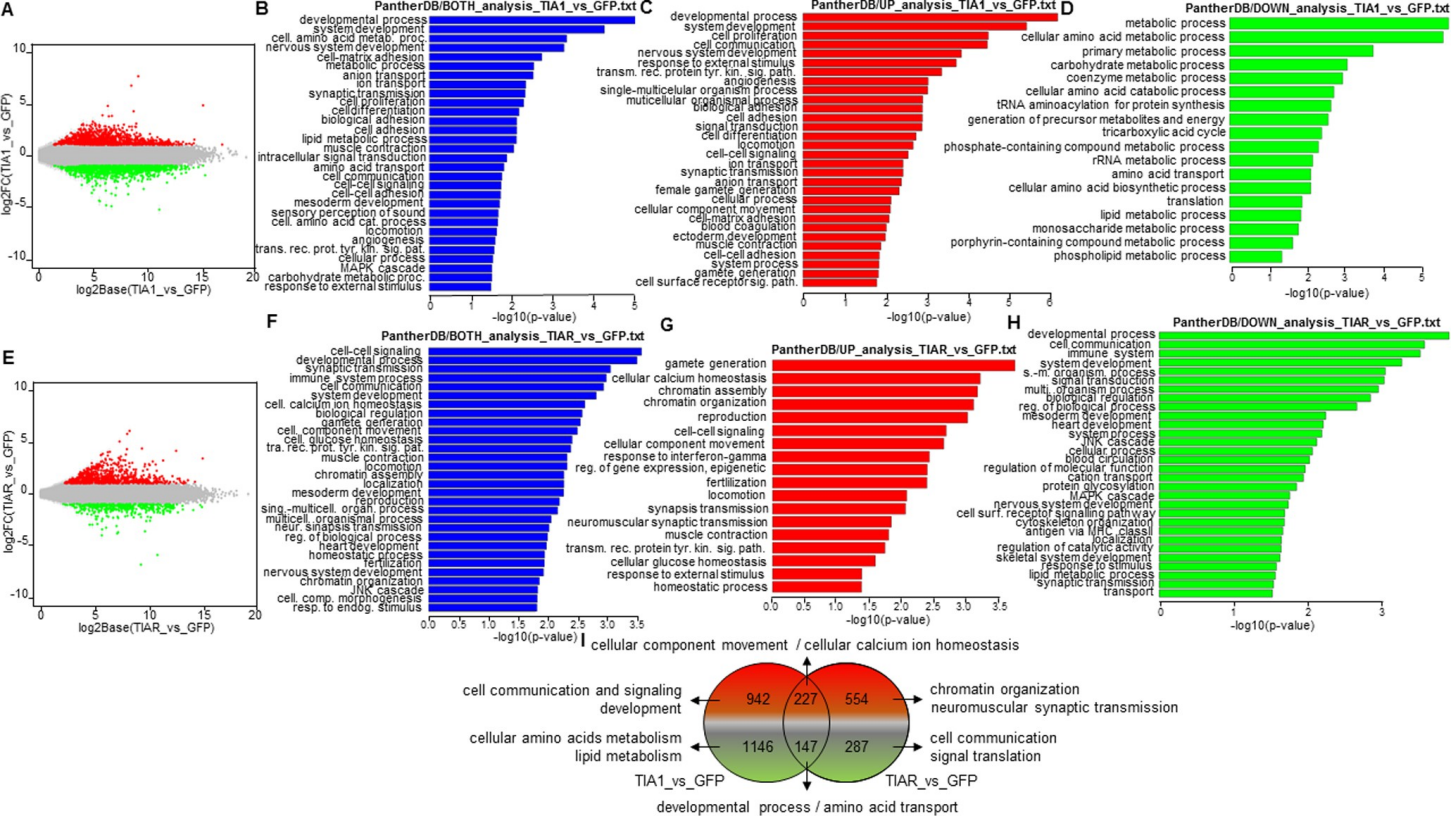
Gene Ontology (GO) analysis of differentially expressed genes (DEGs) in GFP-, GFP-TIA1b-, and GFP-TIARb-expressing FT293 cells. (A-H) Top categories of biological processes associated with DEGs. (A and E) MA plot representations of the distributions of up- (spots in red) and down- (spots in green) expressed genes (-1 ≥ log fold change ≤ 1; FDR < 0.05) in the corresponding combinations indicated in A and E panels, respectively. (B-D and F-H) Histograms of the distributions of up- and down-regulated genes (B and F), only up-regulated genes (red in C and G) and only down-regulated genes (green in D and H) in the corresponding combinations using the GO PANTHER database (*P* < 0.05). (I) Venn diagram displaying numbers and GO enriched categories of DEGs in TIA1- and TIAR-expressing FT293 cells compared with control (GFP-expressing FT293 cells).

As shown in Fig 2, TIA1b and TIARb expression resulted in a marked alteration in gene expression compared with that of GFP-expressing cells. MA plots generated with the FIESTA tool were used to illustrate these analyses, showing up- and down-regulated genes for TIA1b (Fig 2A) and TIARb (Fig 2E). A total of 2,462 (of which 1,169 and 1,293 were up- and down-regulated, respectively) and 1,215 (of which 781 and 434 were up- and down-regulated, respectively) RNAs were differentially expressed (FDR < 0.05) in GFP-TIA1b and GFP-TIARb expressing cells, respectively (Fig 2A and 2E, and S2 Fig). To cluster differentially expressed genes (DEGs) and identify relevant biological processes associated with the ectopic expression of RBPs, transcripts were analyzed with PANTHER (http://pantherdb.org). Among the Gene Ontology (GO) categories identified from the sum of significantly over- and down-expressed genes in TIA1b- *versus* GFP-expressing cells were processes associated with development, cellular amino acid metabolism, nervous system, cell-matrix adhesion, anion and ion transport, synaptic transmission, cell proliferation and differentiation, lipid metabolism, muscle contraction, intracellular signal transduction, amino acid transport, cell communication, cell-cell signaling and adhesion, sensory perception of sound, cell amino acid catabolism locomotion, angiogenesis, transmembrane receptor protein tyrosine kinase signaling and MAPK pathways, carbohydrate metabolism, female gamete generation, heart development, tricarboxylic acid cycle, coenzyme metabolism, synaptic vesicle exocytosis, tRNA aminoacylation for protein translation and phosphate-containing compound metabolism (Fig 2B). The GO categories identified from differentially up-regulated genes involved processes related to nervous system development, cell proliferation and communication, transmembrane receptor protein tyrosine kinase signaling pathway, angiogenesis, cell adhesion and differentiation, locomotion, cell-cell signaling, female gamete generation, cell-matrix adhesion, blood coagulation, cell-cell adhesion, gamete generation, anatomical structure morphogenesis, cytokine production, MAPK cascade, skeletal system development and cell growth (Fig 2C). By contrast, the categories with differentially down-regulated genes were linked to important aspects of cell metabolism including amino acids, carbohydrates, coenzymes, tRNA aminoacylation for protein translation, generation of precursor metabolites and energy, tricarboxylic acid cycle, ribosomal RNA metabolism, amino acid transport and biosynthesis, lipids, porphyrin and phospholipids (Fig 2D). Collectively, these findings suggest that short-term expression of TIA1b supports cellular processes linked to development and membrane signaling dynamics, and restrains several essential processes of cellular metabolism and translation.

On the other hand, GO terms related to significantly overexpressed and repressed genes in TIAR *versus* GFP-expressing cells involved cell-cell-signaling, development, synaptic transmission, immune system, cell communication, cell calcium ion homeostasis, gamete generation, transmembrane receptor protein tyrosine kinase signaling pathway, muscle contraction, locomotion, chromatin organization and assembly, reproduction, neuromuscular synaptic transmission, JNK and MAPK cascades, monosaccharide metabolism, macrophage activation, signal transduction, response to interferon-gamma, neuron-neuron synaptic transmission, blood circulation, and cell proliferation and differentiation (Fig 2F). The GO terms identified from differentially up-regulated genes involved processes related to gamete generation, cell calcium ion homeostasis, chromatin assembly and organization, reproduction/fertilization, cell-cell signaling and component movement, response to interferon gamma, epigenetic regulation, locomotion, neuromuscular synaptic transmission, muscle contraction, transmembrane receptor protein tyrosine kinase signaling pathway, glucose homeostasis and homeostatic stimulus and process (Fig 2G). Whereas, the GO terms with differentially down-regulated genes were linked to important aspects of development, cell communication, imnune system, JNK and MAPK cascades, protein glycosylation, blood circulation, cytoskeleton organization, antigen processing, skeletal system, lipid metabolic process, synaptic transmission, macrophage activation, endocytosis, nitric oxide mediated signal transduction, cell death and calcium-mediated signaling and carbohydrates metabolism (Fig 2H). These findings suggest that short-term expression of TIARb favors cell processes linked to gametes, reproduction, fertilization, chromatin organization, cell-cell signaling, synapsis and muscle transmission, and restrains several essential processes of cell development, cell communication, immune system, and intracellular signaling. These changes are collectively summarized in Fig 2I, showing a degree of overlap between the biological functions regulated by the two RBPs. Overall, these observations indicate that TIA1b and TIARb regulate both specific and overlapping aspects of the human transcriptome, suggesting that their functional roles can be exclusive, independent, or concurrent, in agreement with our previous observations [33, 47].

### Functional analysis of differentially expressed genes in HuR-expressing FT293 cells

**Fig 3:**
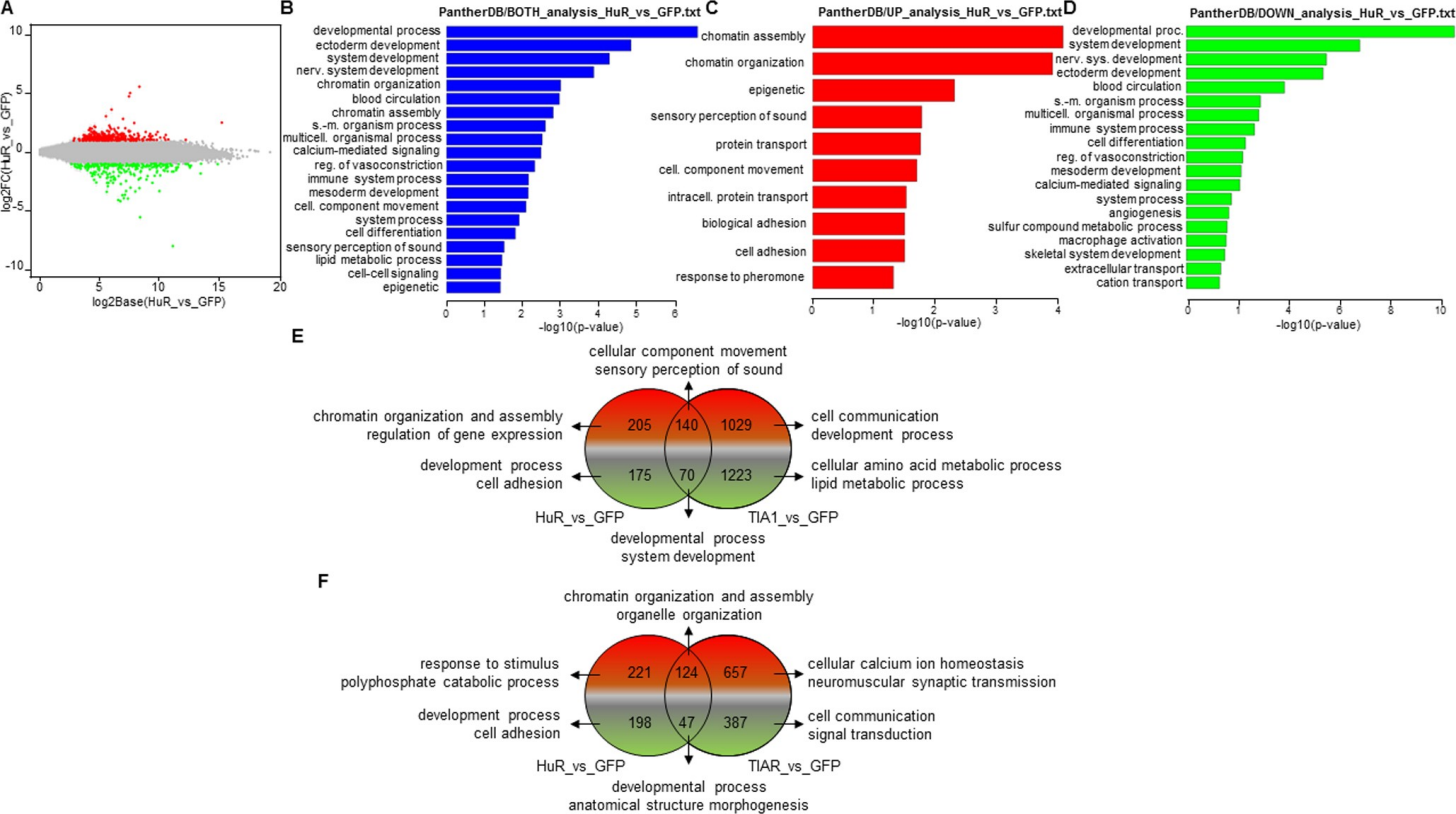
Gene Ontology (GO) analysis of differentially expressed genes (DEGs) in GFP- and GFP-HuR-expressing FT293 cells. (A-D) Top categories of biological processes associated with DEGs. (A) MA plot representations of the distributions of up- (spots in red) and down- (spots in green) expressed genes (-1 ≥ log fold change ≤ 1; FDR < 0.05) in the corresponding combinations indicated in A panel. (B-D) Histograms of the distributions of up- and down-regulated genes (B), only up-regulated genes (red in C) and only down-regulated genes (green in D) in the corresponding combinations using the GO PANTHER database (*P* < 0.05). (E and F) Venn diagrams displaying numbers and GO enriched categories of DEGs in the comparisons HuR *vs* GFP against TIA1 *vs* GFP (E) and HuR *vs* GFP against TIAR *v*s GFP (F) combinations.

A total of 590 (of which 345 and 245 were up- and down-regulated, respectively) RNAs were differentially expressed (FDR < 0.05) in GFP-HuR *versus* GFP-expressing cells (Fig 3A and S2 Fig). GO terms related to significantly overexpressed and repressed genes in HuR *versus* control cells included developmental processes (ectoderm, nervous system an mesoderm), chromatin organization and assembly, blood circulation, calcium-mediated signaling, regulation of vasoconstriction, immune system, cell differentiation, lipid metabolism and cell-cell signaling (Fig 3B). GO terms containing up-regulated genes included epigenetic regulation (chromatin assembly and organization), sensory perception of sound, protein transport, cellular component movement, intracellular protein transport, biological and cell adhesion and response to pheromone (Fig 3C). GO terms involving down-regulated genes included developmental processes (nervous system, ectoderm and mesoderm), blood circulation, immune system, cell differentiation, regulation of vasoconstriction, calcium-mediated signaling, angiogenesis, macrophage activation, skeletal system development and extracellular and cation transport (Fig 3D). Together, these observations suggest that controlled HuR expression favors epigenetic regulation, protein intracellular transport and cell adhesion, whereas it restrains developmental processes and the immune system. The overall trends detected in the above results were confirmed for the TIA1b *versus* HuR (Fig 3E) and TIARb *versus* HuR (Fig 3F) comparisons.

### Classification and splicing analysis of differentially expressed genes

**Fig 4:**
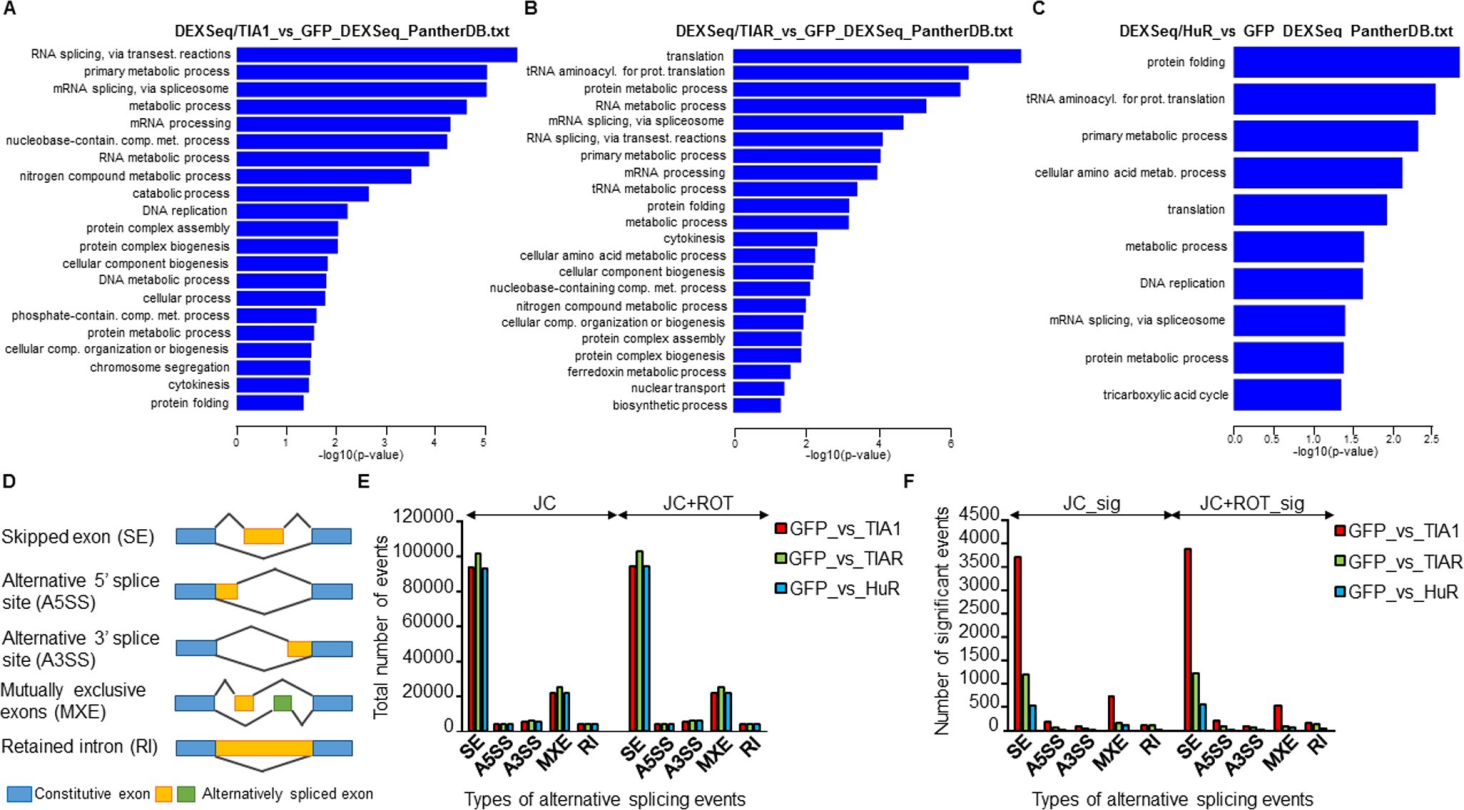
Gene Ontology (GO) analysis of differential splicing events (DSEs) in RBP-expressing FT293 cells. (A-C) Top categories of biological processes associated with DSEs using the GO PANTHER database (*P* < 0.05). Histograms of the distributions of DSEs in the pairs TIA1 *vs* GFP (A), TIAR *vs* GFP (B) and HuR *vs* GFP (C) are shown. (D) Scheme and legends of differential alternative splicing events (ASEs) detected by multivariate analysis of transcript splicing (MATS) taken from Xing lab (http://rnaseq-mats.sourceforge.net). (E and F) Bar plots represent the number of each type of alternative splicing event detected using MATS. (E) JC: total number of events detected using junction counts only. JC+ROT: total number of events detected using both junction counts and reads on targets. (F) JC_sig and JC+ROT_sig represent number of significant events in the above categories, respectively.

We next examined differential exon usage (DEU) using the DEXSeq algorithm [36]. Accordingly, DEU for GFP *versus* TIA1b, TIARb, or HuR data was calculated and the DEU ratios were tested for statistical significance (-1 ≥ log fold-change ≥ 1; FDR < 0.05). A summary of the number of DEU events is shown in Fig 4A–4C and S3 Fig. The corresponding comparisons between TIA1b, TIARb and HuR *versus* GFP resulted in 89 (56 singles), 89 (64 singles), 57 (36 singles) annotated genes, respectively (S2 Fig). GO terms related to DEU (Panther, *P* < 0.05) in TIA1b- *versus* GFP-expressing cells included RNA metabolic processes (RNA and mRNA splicing and nucleobase-containing compound metabolic process), DNA replication, cell communication, and protein and catabolic metabolism (Fig 4A). In the same vein, GO terms related to DEU (Panther, *P* < 0.05) in TIAR *versus* GFP-expressing cells involved protein (translation and tRNA aminoacylation for protein translation) and RNA (RNA and mRNA splicing) metabolic processes (Fig 4B). Finally, GO terms related to DEU (Panther, *P* < 0.05) in HuR *versus* GFP-expressing cells were associated with protein folding, tRNA aminoacylation for protein translation, cellular amino acids metabolic process, translation and DNA replication (Fig 4C). Taken together, these observations suggest that TIA1b and TIARb have a prevalent influence on splicing and translational events, whereas HuR impacts protein metabolism.

Next, to know whether genes with DEU could be directly targeted by TIA1, TIAR or HuR, we compared these genes with human genes containing *in vivo* binding sites for these RBPs substantiated from experimental TIA1/TIAR [6] and HuR [11, 12] individual-nucleotide resolution UV crosslinking and immunoprecipitation (iCLIP) methods. We observed a representative percentage (39%, 60%, and 40%) of potential targets in all the DEU gene categories compared (S3 Fig). In addition, when the genes identified using this filter were clustered by GO analysis, the results indicated that the biological processes agreed with the previous ones. Thus, prevalent GO categories were associated with nucleobase-containing compound metabolic process, RNA and mRNA splicing, and DNA metabolic processes by TIA1b; RNA splicing and protein (translation) metabolic processes by TIARb; and protein folding together RNA and protein metabolic processes by HuR (S3 Fig). These results suggest that many genes identified using the above analysis could be direct targets of these RBPs.

Additionally, multivariate analysis of transcript splicing (MATS) is a useful computational tool to detect differential alternative splicing events (ASEs) from RNA-seq data [41–43]. This procedure automatically detects and analyzes ASEs that correspond to the main categories of known alternative splicing patterns, such as skipped exons (SE), alternative 5' site use (A5SS), alternative 3' site use (A3SS), mutually exclusive exons (MXE), and retained introns (RI) (Fig 4D). For each of the paired samples (i.e., GFP *vs* TIA1b, GFP *vs* TIARb and GFP *vs* HuR), we calculated the number of ASEs corresponding to each of the above categories. The results were categorized as the total number of events detected using junction counts only (JC) (Fig 4E, left) or both junction counts and reads on target (JC+ROT) (Fig 4E, right), as well as the number of significant events detected using junction counts only (JC_sig) (Fig 4F, left) or both junction counts and reads on target (JC+ROT_sig) (Fig 4F, right). The results revealed a marked number of ASE events associated with each category examined, suggesting that TIA1b > TIARb > HuR expression has a degree of higher incidence on regulation/modulation of alternative splicing (Fig 4F). GO analysis of these categories suggested that TIA1b/TIARb-mediated ASEs were associated with cell death and survival, cellular assembly and organization, cell cycle (G1/S checkpoint regulation), cell signaling, protein synthesis, amino acid metabolism and lipid metabolism, whereas HuR-mediated ASEs were linked to cellular growth and proliferation, cell death and survival, hematological disease, protein synthesis, vitamin and mineral metabolism, cell morphology, cell signaling, cell cycle, DNA replication, recombination, and repair, cellular development, and cell metabolism. Nevertheless, further analysis will be required to confirm/validate the ASEs identified.

### Short-term expression of TIA1b and TIARb decreases cell proliferation and leads to progressive cell cycle arrest in G1/S phase

**Fig 5:**
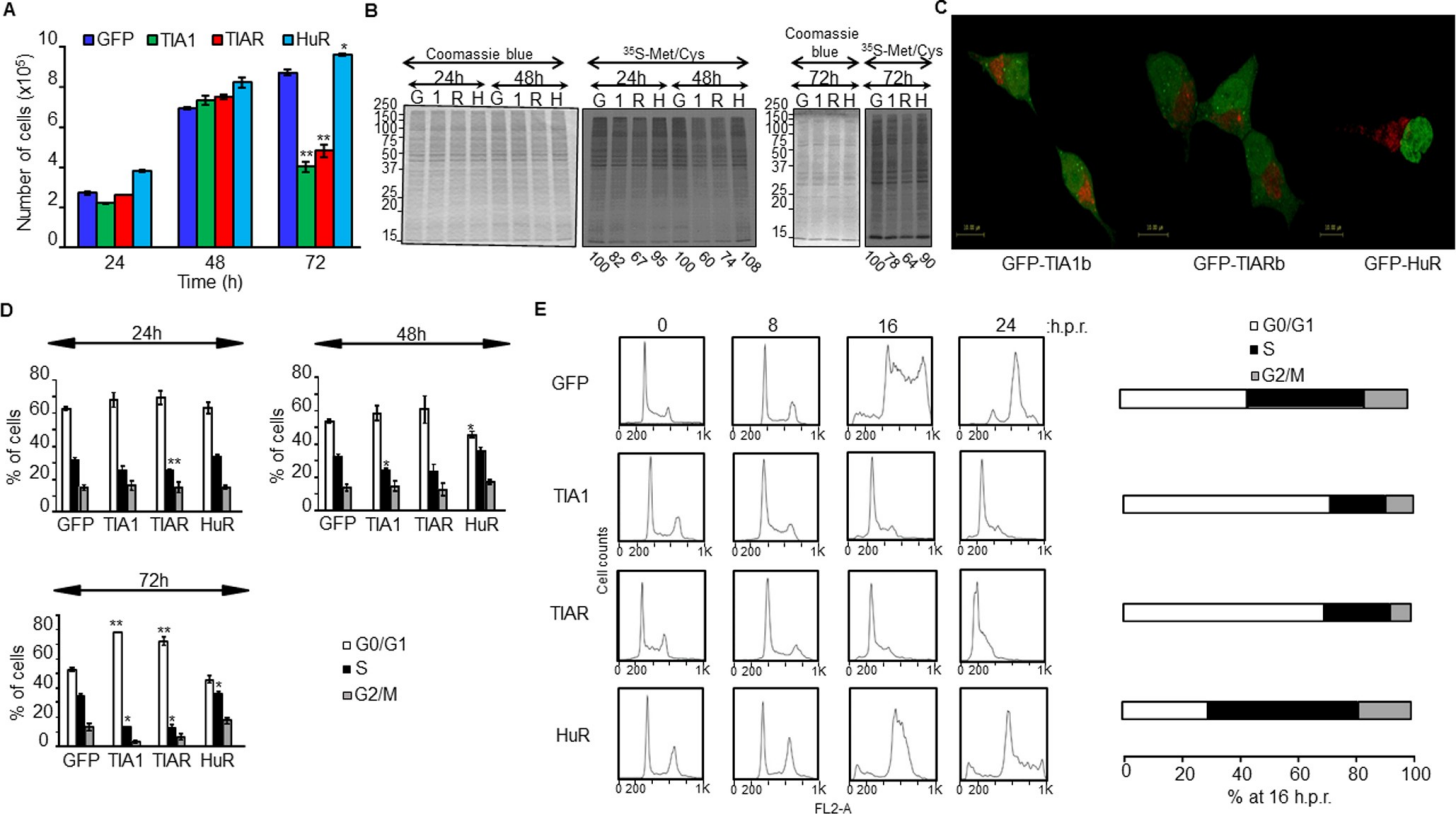
Short-term effect of ectopic RBP expression on rates of cell proliferation, relative translation, subcellular localization, and cell cycle. (A) Rates of cell proliferation. Cells were grown in culture medium containing glucose (10 mM) and counted at 24–72 h. Values indicate means ± SEM (*n* = 3; *P < 0.05; **P < 0.01). (B) Rates of global translation. Translational rates of total proteins in above cells for 24–72 h were determined in the presence of ^35^S-methionine/-cysteine (^35^S-Met/Cys). The relative translational rates were estimated as ratio ^35^S-Met/Cys label *versus* Coomassie Blue reagent staining. The values are indicated as percentage relative to control sample (GFP). The legend identified as G, 1, R, and H indicates GFP, TIA1b, TIARb, and HuR samples, respectively. (C) Rates of subcellular localization. The above cell lines were cultured for 48 h, stained with Mitotracker and visualized by confocal microscopy. GFP-tagged proteins and mitochondria are visualized in green and red, respectively. Scale bars represent 10 μm. (D) Analysis of cell cycle phases by flow cytometry. The percentage of indicated cells was quantified in every cell cycle phase for each experimental condition indicated. Values represent means ± SEM (*n* = 3; *P < 0.05; **P < 0.01). (E) The expression of TIA1b and TIARb in FT293 cells drives cell cycle arrest at the G1/S phase. Cell lines were synchronized at G1/S by hydroxyurea blockage for 24 h, released and quantified at 16 h post-release (h.p.r.).

**Fig 6:**
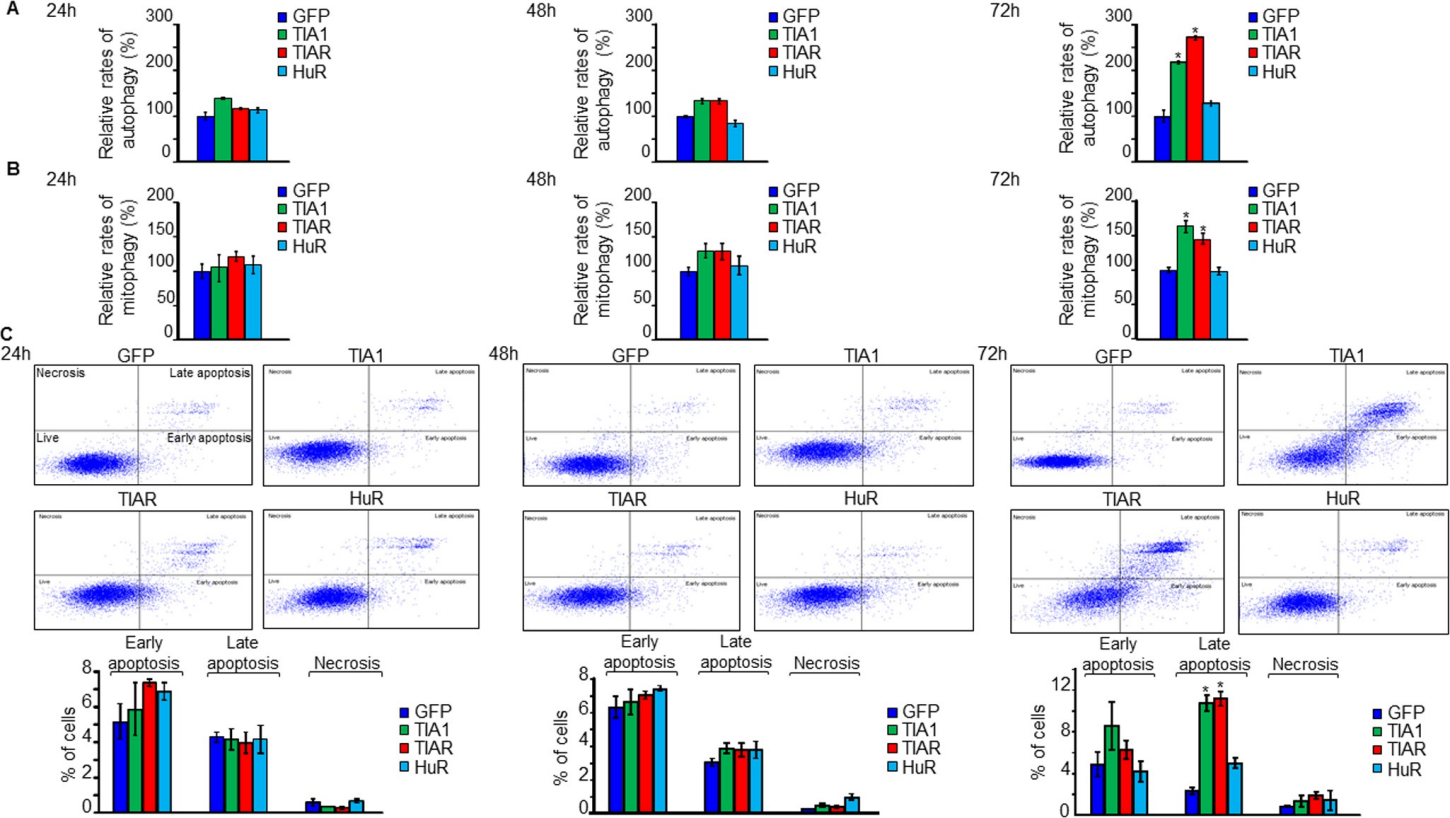
Analysis of autophagy, mitophagy, and apoptosis in RBP-expressing cells for 24–72 hours. (A) Autophagy analysis. Cell lines were transfected with GFP-LC3B-RFP plasmid, visualized by confocal microscopy and quantified. Histograms represent the sum of autophagosomes and autolysosomes estimated as yellow and free red dots per cell, respectively. The represented values were normalized and expressed as percentage relative to the GFP control. (B) Mitophagy analysis using mitochondrial Keima probe. Cell lines were transfected with the mt-Keima-expressing plasmid, visualized by confocal microscopy and counted. Histograms show normalized red/green mt-Keima signals. The represented values were expressed as a percentage relative to the GFP control. (C) Rates of cell death by apoptosis for 3 days. Quantification of apoptosis rates was carried out by flow cytometry. All the represented values are means ± SEM (n = 3; *P < 0.05).

GO analysis of DEGs and DEUs suggested a differential role for TIA1b and TIARb *versus* HuR proteins in the control of biological processes associated with cell proliferation/growth and cell cycle. To functionally test these *in silico* results, we examined the proliferative potential of FT293 cells expressing TIA1b or TIARb at 24–72 hours post-induction. The expression of the RBPs did not significantly compromise cell growth during the first 48 hours after induction; however, a significant reduction in cell proliferation was observed at 72 hours (Fig 5A). By contrast, HuR expression slightly and significantly increased cell proliferation (Fig 5A). Analysis of *de novo* protein synthesis rates by isotopic labeling with a mixture of ^35^S-methionine/cysteine showed a decrease in global translational rates in GFP-TIA1b- and GFP-TIARb-expressing cells (73±7% and 68±3%, respectively) over the three days studied as compared with GFP- and HuR-expressing FT293 cells (99±5%) (Fig 5B). This decrease in protein synthesis in TIA1b and TIARb expressing cells was accompanied by the presence of discrete foci (apparent granule-like structures) (green dots in cytoplasm) together with mitochondrial clustering (red dots in cytoplasm) at 48 hours post-induction (Fig 5C and S4–S6 Figs). Additionally, TIA1b and TIARb expressing cells showed a progressive arrest at the G1/S phase of the cell cycle (Fig 5D), whereas HuR expressing cells showed a significant increase in S and G2/M phases (Fig 5D). This observation was also validated and confirmed by analysis of cell cycle progression under synchronized growth following release from hydroxyurea (Fig 5E). These cellular phenotypes were observed without a compromise in cellular viability upon TIA1b or TIARb expression over 24-48h, with similar rates of autophagy (Fig 6A), mitophagy (Fig 6B) and cell death (Fig 6C). After 72 hours, however, all of these outcomes were significantly higher in TIA1b/TIARb expressing cells than in HuR cells (Fig 6A–6C). Taken together, these observations suggest that the short-term expression of TIA1b and TIARb leads to an early situation of cell stress and quiescence.

### Validation of massive sequencing-predicted changes in gene expression patterns

**Fig 7:**
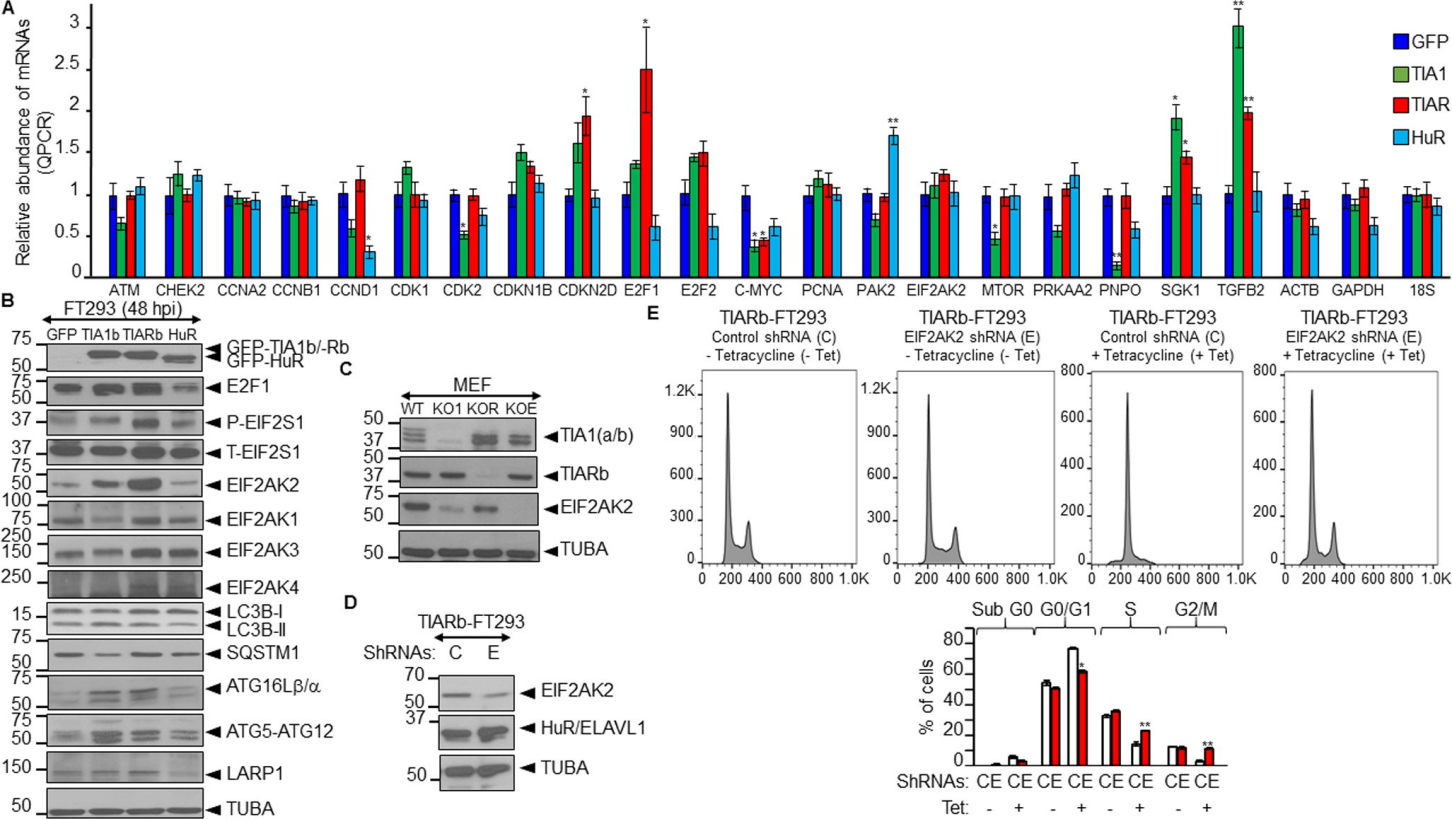
Regulatory crosstalk between the expression of TIA proteins and EIF2AK2-mediated cell quiescence. (A and B) Validation and quantification of relative expression levels of RNAs and proteins by qPCR (A) and western blot (B) analysis. Protein extracts and cytoplasmic RNAs were isolated from GFP-, TIA1b-, TIARb-, and HuR-expressing FT293 cells and analyzed by qPCR (A) and western blotting (B). The represented values were normalized and expressed relative to the GFP control (A). The values are means ± SEM (n = 2–3; *P < 0.05; **P < 0.01). For western blot analysis were used the antibodies indicated. (C) Effect of the disruption of mouse TIA1, TIAR and EIF2AK2 genes on TIA1, TIAR and EIF2AK2 expression in murine embryonic fibroblasts (MEFs). Protein extracts from normal (WT MEF) or knocked out for TIA1 (KO1 MEF), TIAR (KOR MEF) or EIF2AK2 (KOE MEF) were purified and processed for western blot analysis using the antibodies indicated. (D) EIF2AK2 knockdown using lentiviral particles in TIARb-expressing FT293 cells. Western blot analysis of protein extracts of TIARb-expressing FT293 cells (without tetracycline means in the absence of TIARb expression) stably transfected with shRNAs against control (*C*) and EIF2AK2 (*E*) using a lentiviral transfection. The blot was probed with specific antibodies against EIF2AK2, HuR/ELAVL1 and TUBA proteins. (E) TIARb-expressing FT293 cells transfected with shRNAs against control (*C*) and EIF2AK2 (*E*) were seeded and grown for 3 days in the absence (-) and presence (+) of tetracycline. Above cells were monitored by flow cytometry after propidium iodide staining. The data are means ± SEM of the three independent experiments (n = 3; *P < 0.05; **P < 0.01). (B-D) Molecular weight markers for protein (kDa) and the identities of protein bands are indicated on the left and right by arrowheads, respectively.

We next validated the RNA-seq data by quantitative RT-PCR assays for 19 differentially expressed genes involved in cell-cycle, translation, metabolism and autophagy. As shown in Fig 7A, expression of TIA1b, TIARb or HuR altered the steady-state levels of the analyzed mRNAs. These results also indicate that the actions of TIA1b, TIARb and HuR proteins can be independent, additive, overlapping and/or antagonistic. In principle, the results agreed with the less proliferative phenotypes and progressive arrest in G1/S phase observed in the TIA1b and TIARb expressing cells. These results also suggest the existence of a compensatory mechanism on cell-cycle components mediated by the master transcription factor E2F1; although we have not detected gene signatures associated to E2F1-dependent transcription (Fig 7A). We extended this validation using western blotting to measure the expression of some proteins linked to relevant biological processes or pathways including the cell-cycle (E2F1), translation (EIF2S1 and EIF2AK1-4) (S7 and S8 Figs), and autophagy/proteostasis (LC3B, SQSTM1, ATG16L, ATG5-ATG12 and LARP1) (Fig 7B). The results were partly consistent with those of the RNA-seq analysis, suggesting that some changes observed in our transcriptomic data could represent putative novel target genes regulated by TIA1b, TIARb or HuR proteins.

To get new insights into the regulatory crosstalk between of TIA proteins and EIF2AK2 expression, we used western blotting to measure their relative protein expression in murine embryonic fibroblasts (MEFs) knocked-out for TIA1 [26], TIAR [27], or EIF2AK2 [32]. The results indicated that EIF2AK2 expression was decreased in TIA1 and TIAR KO MEFs (Fig 7C), suggesting that these RBPs can modulate the expression of EIF2AK2, confirming previous results [48]. In addition, we questioned us whether EIF2AK2 expression detected in TIA-expressing FT293 cells could be directly contributing to the cell-cycle arrest at the G1/S phase observed in above cells. To test this possibility, we generated a new TIAR-expressing FT293 cell line with down-regulated expression of EIF2AK2 by using control and EIF2AK2 shRNA-expressing lentivirus. Our results show a partial reduction (60%) of EIF2AK2 expression (Fig 7D). These TIAR-expressing FT293 cells with either normal or downregulated expression of EIF2AK2 were grown in the absence and presence of tetracycline for 3 days to induce GFP-TIARb expression (Fig 7E). The FACS analysis in above FT293 cells showed that the cell-cycle arrest at the G1/S phase observed in TIARb-expressing FT293 cells was significantly reverted to S and G2/M phases under the EIF2AK2 downregulation (Fig 7E). Taken together, these observations suggest that gene expression patterns and cellular phenotypes associated with TIA-expressing FT293 cells are partially reverted in an EIF2AK2-dependent way.

## Discussion

**Fig 8:**
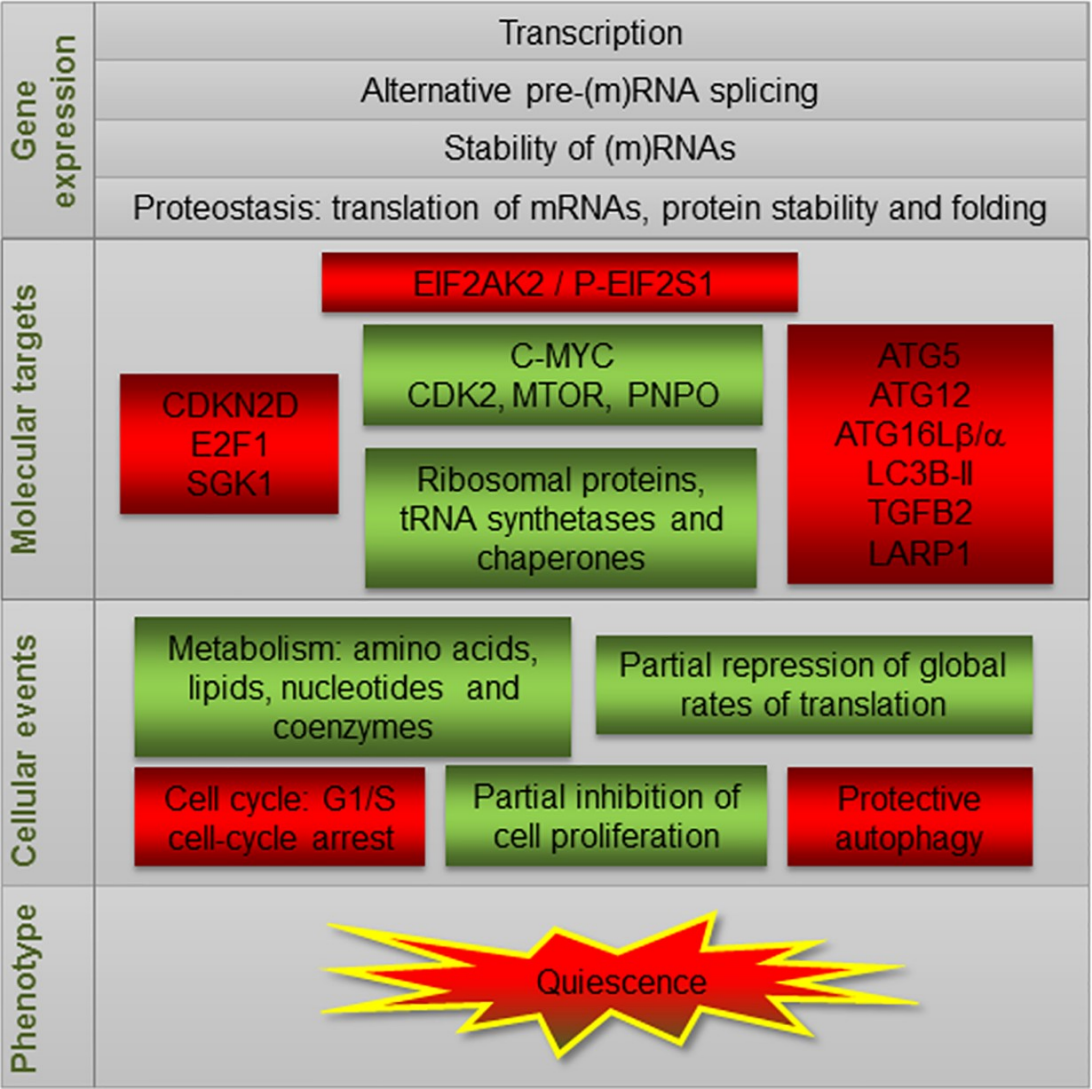
A working model summarizing the molecular and cellular events associated with the ectopic short-term expression of TIA proteins.

To reveal functional and molecular events operating in RBP-expressing cells, we performed a global transcriptome analysis to identify DEGs and differential splicing events (DSEs) in HEK293 cells. Our observations indicate that ectopic expression of TIA1b and TIARb alters the transcriptome and decreases cell proliferation, global protein synthesis and cell cycle transition at G1/S. To our knowledge, this is the first transcriptome-wide analysis to uncover DEGs and DSEs that are early induced by TIA1b, TIARb, or HuR expression. We anticipate that these data will be a powerful resource for future investigations into the effects of TIA and HuR on survival and/or quiescence-associated responses (Fig 8).

RBPs have major effects on all aspects of cellular RNA fate and metabolism through their function as cell executors and sensors to maintain homeostasis and aid cell adaptation to a range of stressing clues involving short-term environmental challenges. To coordinate these responses, these polyvalent regulators shape transcriptional and post-transcriptional dynamics of RNA collections (regulons) of differentially expressed RNAs and spliced RNA variants, which condition biological programs related to development, proliferation/differentiation, cell cycle, translation, autophagy and apoptosis [7, 8]. Herein, we report that TIA1b and TIARb overexpression has a prevalent impact on regulatory aspects associated with development, metabolism, and proteostasis. The biological consequences associated to the short-time HuR expression were attenuated, observing a limited effect on cell proliferation and cell-cycle linked to epigenetic regulation, protein intracellular transport and cell adhesion, whereas it restrains developmental processes and the immune system. These observations agree with the best-known functions of HuR and their key role as post-transcriptional master regulator [8–16, 49].

Our results show that either TIA1b, TIARb, or HuR expression regulates the transcriptomes of both embryonic and specific tissue development. Exploring TIA1 and TIAR function *in vivo* is challenging since their constitutive knockout is lethal [26, 27], and mice lacking both of these proteins die before embryonic day 7, implying that at least one of these proteins must be present for normal embryonic development [26]. Whereas targeted ablation of TIA1 [26] or TIAR [27] results in high embryonic lethality, the penetrance varies between TIA proteins: close to 100% and around 50% in TIAR-knockout mice on BALB/c and C57Bl/6 backgrounds, respectively and 50% in TIA1-knockout mice on the BALB/c background, whereas normal litter sizes for TIA1 null mice occur on the C57Bl/c background [50]. The reason for the variable penetrance of TIA1 inactivation in BALB/c mice is unknown. Further, whereas murine nullizygotes of TIA1 are fully fertile, mice lacking TIAR are sterile due to a specific requirement for TIAR in primordial germ cell development and survival [27]. Indeed, the phenotype of surviving adult TIAR-knockout mice displays decreased embryo size, absent ovarian follicles, decreased primordial germ cell number, small ovary and testis, ovary hyperplasia, and postnatal growth retardation [27]. By contrast, TIA1-depleted adult mice present middle-age arthritis, abnormal macrophage physiology and increased susceptibility to endotoxin shock, but with normal hematopoietic and immune system phenotypes [26]. However, it has been recently shown that TIA1 is also a conditional gender-specific disease modifier in a mild mouse model of spinal muscular atrophy, because it enhances the impairment of male reproductive organ development and exacerbates the gene expression of the testis-specific transcriptome [50]. The deletion of TIA1 in this mouse model appears to disrupt many physiological processes within the testis. Further, overexpression of TIAR in mice impairs embryonic development at late preimplantation and post-implantation stages, suggesting that balanced TIAR expression is required for early embryogenesis [28]. Overall, genetic studies have demonstrated both shared and unique characteristics of these closely related proteins. In the same vein, targeted disruption of HuR drives lethality within 10 days of postnatal life [29]. Therefore, it is difficult to determine the *in vivo* primary roles of TIA and HuR proteins using traditional genetic approaches. For these reasons, the precise molecular mechanisms through which these proteins regulate developmental and cellular processes are largely unknown, and the generation of new mouse models with inducible developmental- and/or tissue-specific ablation of these genes will be needed to precisely define their roles.

Our studies suggest that the nervous system is targeted by the expression of TIA proteins. Consistent with this notion is the recent demonstration that loss of TIA1 in female mice impacts autonomic nervous system input to negatively affect the tail vasculature, perhaps through reduced blood flow and/or accelerated blood vessel degeneration [50]. Thus, TIA1 ablation differentially affects gene expression in brain of males and females, suggesting that TIA1 may serve as a gender-specific modifier of several neurological diseases such as ALS, Alzheimer’s disease and frontotemporal lobar dementia [50]. Indeed, emerging studies have shown that TIA1 plays a key role in mediating *in vivo* toxicity, tau-aggregation and tau-mediated pathology [19, 20] as well as aberrant formation and dynamics of stress granules, which drives the generation of pathological stress granules [19–21]. Furthermore, TIA1 expression may be a better marker of obesity in females than in males, because downregulation of several genes in TIA1-knockout female brain may contribute to body weight gain, as the nervous system partly controls body weight [50]. Strengthening this notion, a previous study showed that TIA1 is one of the top-ranking genes downregulated in obese patients [51]. Moreover, a study of the transcriptome in spinal cord and cerebellum of TIA1-knockout mice [26] described a major effect on mRNAs encoding lipid homeostasis factors in the brain, similar to a fasting effect [52]. Due to the conserved protein structure architecture between TIA1 and TIAR, most of the TIA1-associated functions are expected to be performed by TIAR. Indeed, adult TIAR-knockout mice also present an obesity-related phenotype [27]. These results, together with the present study and our recent observations showing the functional antagonism between TIA and HuR proteins in regulating mitochondrial activity and dynamics [18], suggest that several metabolic aspects are targeted by TIA expression, such as the biosynthesis of carbohydrates, lipids, amino acids, and nucleotides (e.g., folate coenzyme).

Obesity is associated with susceptibility to several pathologies, including tumorigenesis and it favors both increased cancer risk in patients and onset of disease in pre-clinical models [53]. In fact, obesity now rivals smoking as one of the leading preventable causes of oncogenesis; however, it remains unclear whether obesity-associated inflammation promotes cancer progression and metastasis. It has been appreciated for some time that chronic inflammation, which can be driven by tumors or other pathophysiological conditions, creates favorable conditions for metastases and growth [53]. Our results and those of others suggest that a molecular link could exist between aging-dependent aberrant expression of TIA1 and/or TIAR and obesity-associated deleterious phenotypes [54, 55]. Further, TIA expression alters mitochondrial dynamics and function [18] and there is a strong link between adult-onset obesity and mitochondrial dysfunction [56, 57]. Changes in mitochondrial function have long-term consequences for energy metabolism and can be a major predisposing factor for the development of the metabolic syndrome. By contrast, HuR expression is related to cellular and molecular events associated with chromatin dynamics. The substrates used to modify nucleic acids and chromatin are affected by nutrient availability and the activity of metabolic pathways [18, 49]. Thus, cellular metabolism constitutes a fundamental component of chromatin status and genome regulation [49, 58].

Alternative and constitutive splicing are post-transcriptional events prevalently associated with TIA1 and TIAR expression. mRNA precursors (pre-mRNAs) and mRNAs associate with regulatory factors, and the combinatorial binding of RBPs and/or miRNAs to mRNA targets co-ordinate the different steps of the RNA life cycle [7, 8]. TIA proteins facilitate splicing of pre-mRNAs by enhancing the selection of constitutive and atypical 5' splice sites [59–61]. Pre-mRNAs whose splicing is regulated by TIA proteins contain specific sequences close to the intron 5’-splice sites, composed mainly by U-rich stretches [6, 59–61]. It will be important to determine whether ectopic or endogenous TIA proteins, as well as potential post-translational modifications of these factors, can also regulate the gene expression patterns of these target mRNAs at transcriptional and/or post-transcriptional levels. Against this background, our observations suggest a direct interaction between some mRNAs and TIA proteins, which could contribute to the post-transcriptional regulation—alternative pre-mRNA splicing, mRNA turnover/stability and/or mRNA translation—of these gene targets. Accordingly, RBPs are able to function as global/specific translational repressors, possibly by contributing to plasticity in growth and metabolism that enables cells and tissues to adapt to a dynamic environment [62, 63]. This suggests that TIA proteins could regulate differential subsets of RNAs at the splicing and translational levels. The available TIA-UV cross-linking and immunoprecipitation (iCLIP) data have expanded the total number of post-transcriptional events and associated gene functions that could be predicted to be regulated by TIA proteins. Thus, our results suggest that the estimated frequency of regulatory post-transcriptional events modulated by TIA1/TIAR *via* specific-sequences motifs may be 10–20%, in agreement with previous observations [6]. In the same vein, HuR facilitates constitutive and alternative splicing, stability, and polyadenylation regulatory events [8–16]. For example, iCLIP analysis have shown that a high proportion (10–15%) of HuR binding sites are located on introns [11, 12]. Thus, HuR modulates the speed of transcription because can interacts with RNA pol II, histone deacetylase II (HDAC2) and nascent RNAs, directly improving constitutive and alternative splicing of targeted genes and other splicing factors [8, 11, 12, 64].

The molecular mechanism of TIA-induced translational inhibition has been partially characterized for conditions of environmental stress involving oxidation and nutrient deprivation responses. Thus, under oxidative-stress conditions, cytoplasmic protein biosynthesis is regulated by phosphorylation of the alpha subunit of the eukaryotic translation initiation factor 2 (EIF2S1/eIF2alpha) at serine 51 [65, 66]. Four distinct stress-sensing serine/threonine protein kinases have been described in humans that inhibit translation initiation at the 5’UTR of mRNAs *via* impairing the assembly of the ternary eIF2/tRNAiMet/GTP complex. Accordingly, EIF2AK1/HRI responds to heme deprivation, oxidative stress and heat shock. EIF2AK2/PKR is induced by innate immunity/viral infection and nutrient and organelle dysfunction. EIF2AK3/PERK is activated by unfolded proteins in the endoplasmic reticulum and responds to metabolic stress, viral infection and hypoxia. EIF2AK4/GCN2 is sensitive to amino acid starvation, UV irradiation, proteasome inhibition and viral infection [67, 68]. TIA proteins contribute to the formation and assembly of unproductive translational pre-initiation complexes containing mRNAs and proteins *via* the generation of stress granules [65, 66, 69]. For example, TIA1, through oxidation of cysteine 36, contributes to cell survival not only by suppressing translation but also by sequestering some apoptosis regulatory proteins. Thus, cellular protein misfolding together with reactive oxygen species-induced oxidative stress promotes oxidation of TIA1, and its aggregation, and it cannot form stress granules to trigger apoptosis [70]. This notion is relevant because this mechanism can be operating in neurodegenerative diseases [18–20]. Another example is amino acid starvation: TIA proteins are tethered onto the 5′ end of 5′-terminal oligopyrimidine tracts (5′-TOP) mRNAs to arrest translation at the initiation step. The TIA-dependent 5′-TOP mRNA translation repression implies polysomal release and accumulation in stress granules [71]. This repressor activity requires starvation-mediated activation of the GCN2 kinase, which can be activated by the accumulation of unchanged tRNAs, and inactivation of the mTOR signaling pathway [72, 73]. These observations could explain how large networks of cellular RNAs encoding protein biosynthesis factors can be post-transcriptionally co-regulated in response to distinct environmental-stress conditions by specific RBPs such as TIA proteins (S7 Fig).

A recent paper using HEK293 cells suggested that human TIA proteins exert their redundant functions by contributing to the fidelity of pre-mRNA splicing and mRNA turnover through targets encoding proteins required for the regulation of cell cycle progression and EIF2AK2 activation in a cell-specific manner [48]. We have recently shown that ectopic TIA1b or TIARb expression in FT293 cells promotes the depletion of endogenous TIA1 and TIAR proteins, respectively [17]. This depletion occurred in a ΔQ-domain-dependent manner and was associated only with the expression of TIA proteins, as the ectopic expression of TIA1b or TIARb did not alter endogenous HuR expression [17]. In the same way, endogenous expression of TIA1 and TIAR was unaffected by ectopic HuR expression, whereas endogenous HuR levels were decreased in a HuR-dependent manner [17]. Moreover, we and others have previously demonstrated that TIA1 or TIAR activate splicing of some unusually used alternative exons on their pre-mRNAs [17, 73]. These novel RNA isoforms contained exons with premature stop codons that, when included, interrupted their open reading frames, resulting in decreased levels of these factors and facilitating their elimination by nonsense-mediated decay mRNA decay. Therefore, ectopically expressed TIA1 and TIAR (and also HuR) proteins function as post-transcriptional auto-regulators to reprogram the expression of endogenous TIA1, TIAR and/or HuR proteins, validating the use of these gain-of-function models to study and analyze their individual contributions to cellular processes and phenotypes [17, 18, 69]. Thus, our results suggest that EIF2AK2 gene is potentially targeted at the post-transcriptional level (i.e. protein and mRNA stability and/or mRNA translational efficiency) by ectopic TIA1b and TIARb expression, and is up-regulated and phosphorylates EIF2S1 at Ser51, to promote partial inhibition of global translation, apparent granule-like structures, and cell-cycle arrest at the G1/S phase (S7 Fig and Fig 8).

Finally, loss- and gain-of-function of TIA1 can trigger autophagic phenotypes [17, 18, 33]. Increasing evidence suggests that some proteins, including annexin A7 [74], as well as long non-coding RNAs, such as FLJ11812/TGFB2-OT1 [75, 76] and MALAT1 [77], regulate autophagy through modulating TIA1 phosphorylation, to suppress the microRNA-mediated deregulation of some autophagic components in vascular endothelial cells [74–76]. Our results are in agreement with this scenario. Further, an unsuspected crosstalk between cellular metabolism (fatty acids), pro-inflammatory signaling (STAT3), innate immunity (EIF2AK2), and translational control (EIF2S1) has been also linked to autophagy response [78]. Here we provide strong evidence to suggest that short-term expression of TIA1b and TIARb impact on the EIF2AK2-mediated autophagy machinery, promoting an early protective autophagy response to safeguard cell survival under the induction of a cell quiescent phenotype [18].

## Conclusions

The RNA-binding proteins TIA (TIA1 and TIAR) and HuR are essential for cell proliferation/differentiation and survival phenotypes. Combining transcriptome-wide, *in silico* and functional analyses, we identify prevalent regulatory determinants of the differential sensitivity of TIA isoforms b and HuR on gene expression regulation. The controlled expression of these RBPs strongly influence the patterns of differential expressed genes and RNA variants preferentially associated with development, reproduction, cell cycle, metabolism, and autophagy. Mechanistically, TIA1b and TIARb isoforms display both common and differential effects on gene expression regulation. Thus, TIA1b generally displays stronger effects on regulatory events associated with splicing, and TIARb more acute effects on translational control-related components. Our study also demonstrates that RNA-seq analysis provides a comprehensive profile of RBP-expressing cells, which has utility as a dynamic method to delineate and infer possible causes associated with molecular environment and cell phenotype observed for RBP-expressing cells. Taken together, the results establish that splicing and translation is a prevalent locus of regulation by these RBPs at short-term. We expect that this information can be used to help design further studies to expand our current knowledge of physiological responses, with relevance for a better understanding of the various mechanisms and complex regulatory networks that influence the control of gene expression by these RBPs.

## Supporting information

S1 Fig Metrical data of massive-scale RNA sequencing analysis. (XLSX)

S2 Fig List of differentially expressed genes in GFP-TIA1b-, GFP-TIARb-, and GFP-HuR-expressing FT293 cells compared with GFP-expressing FT293 cells identified by RNA-Seq. (XLSX)

S3 Fig List of differentially spliced exons in GFP-TIA1b-, GFP-TIARb-, and GFP-HuR-expressing FT293 cells compared with GFP-expressing FT293 cells identified by RNA-Seq. (XLSX)

S4 Fig 3D-reconstitution of GFP-TIA1b- and GFP-TIA1bΔQ-expressing FT293 cells. GFP-TIA1b- and GFP-TIA1bΔQ-expressing FT293 cells cultured for 48 h, stained with Mitotracker and visualized by confocal microscopy. The serial sections were processed using ImageJ to reconstitute the single images from individual FT293 cells expressing GFP-tagged proteins of interest.
(AVI)

S5 Fig 3D-reconstitution of GFP-TIARb- and GFP-TIARbΔQ-expressing FT293 cells. GFP-TIARb and GFP-TIARbΔQ-expressing FT293 cells were cultured and processed as described in S4 Fig.
(AVI)

S6 Fig 3D-reconstitution of GFP-HuR-expressing FT293 cells. GFP-HuR-expressing FT293 cells were cultured and processed as described in S4 Fig.
(AVI)

S7 Fig *In vivo* cross-linking sites (iCLIP) of TIA1 and TIAR proteins at EIF2AK1-4 and EIFS1 genes. The RNA map, corresponding to TIA proteins on indicated genes in HeLa cells, was adapted using the TIA-iCLIP analysis provided by Jernej Ule's laboratory [6]. The histograms show the number of cDNAs that identified each cross-linking site. The localization of target genes on human chromosomes and the exon and intron positions of the human pre-mRNAs are shown. The following genes were used: EIF2AK1/HRI, heme-regulated eukaryotic initiation factor 2 alpha kinase; EIF2AK2/PKR, interferon-inducible double stranded RNA-dependent serine/threonine protein kinase; EIF2AK3/PERK, PRKR-like endoplasmic reticulum kinase; EIF2AK4/GCN2, amino acid insufficiency-regulated eukaryotic translation initiation factor 2 alpha kinase; and EIFS1/eIF2alpha, eukaryotic translation initiation factor 2 subunit alpha.
(TIF)

S8 Fig List of primer pair sequences and antibodies for qPCR and Western blotting analysis used in the study. (XLS)

## Abbreviations

ACTB: beta-actin
ATM: ataxia telangiectasia mutated serine/threonine kinase
CHEK2: checkpoint kinase 2
CCNA2: cyclin A2
CCNB1: cyclin B1
CCND1: cyclin D1
CDK1: cyclin dependent kinase 1
CDK2: cyclin dependent kinase 2
CDKN1A: cyclin dependent kinase inhibitor 1A
CDKN1B: cyclin dependent kinase inhibitor 1B
CDKN2D: cyclin dependent kinase inhibitor 2D
CMYC: myc proto-oncogen protein
E2F1: E2F transcription factor 1
E2F2: E2F transcription factor 2
EIF2AK1-4: eukaryotic translation initiation factor 2 alpha kinase 1–4
GAPDH: glyceraldehyde phosphate dehydrogenase
MTOR: mechanistic target of rapamycin
HuR/ELALV1: human R antigen
PCNA: proliferating cell nuclear antigen
PAK2: p21(Rac1) activated kinase 2
PNPO: pyridoxamine 5'-phosphate oxidase
PRKAA2: protein kinase AMP-activated catalytic subunit alpha 2
SGK1: serum/glucocorticoid regulated kinase 1
TIA1: T-cell intracellular antigen 1
TIAR/TIAL1: TIA1-related/like protein, and TGFB2, transforming growth factor beta 2
18S: ribosomal RNA 18S
