# Supplementary figures and images for: Transcriptome-wide analysis links the short-term expression of the b isoforms of TIA proteins to protective proteostasis-mediated cell quiescence response
Source: PLoS One. 2018 Dec 11;13(12):e0208526. doi: 10.1371/journal.pone.0208526 (PMC6289441; doi:10.1371/journal.pone.0208526)

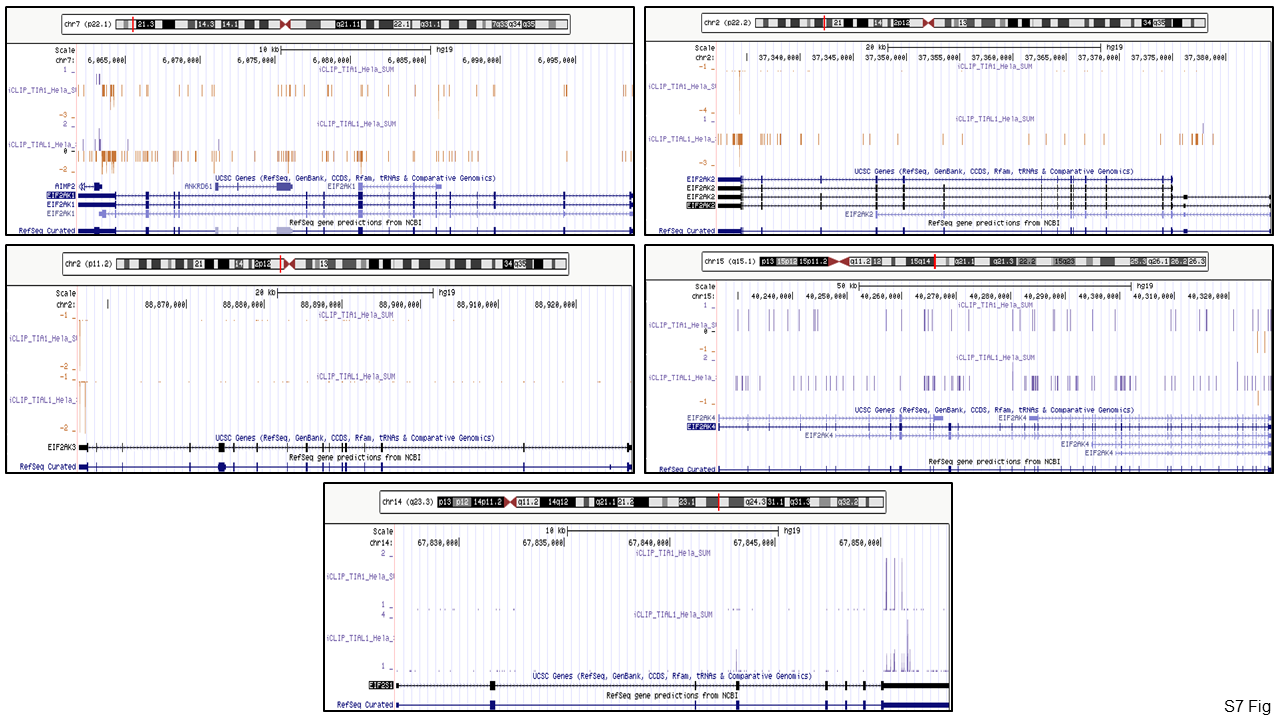

Supplement: S7 Fig — The RNA map, corresponding to TIA proteins on indicated genes in HeLa cells, was adapted using the TIA-iCLIP analysis provided by Jernej Ule's laboratory [6]. The histograms show the number of cDNAs that identified each cross-linking site. The localization of target genes on human chromosomes and the exon and intron positions of the human pre-mRNAs are shown. The following genes were used: EIF2AK1/HRI, heme-regulated eukaryotic initiation factor 2 alpha kinase; EIF2AK2/PKR, interferon-inducible double stranded RNA-dependent serine/threonine protein kinase; EIF2AK3/PERK, PRKR-like endoplasmic reticulum kinase; EIF2AK4/GCN2, amino acid insufficiency-regulated eukaryotic translation initiation factor 2 alpha kinase; and EIFS1/eIF2alpha, eukaryotic translation initiation factor 2 subunit alpha. (TIF) [file pone.0208526.s007.tif]
